# The Impact of an Intensivist-Led Critical Care Transition Program

**DOI:** 10.7759/cureus.21313

**Published:** 2022-01-17

**Authors:** Raul Neto, Margarida Carvalho, Ana Isabel Paixão, Paula Fernandes, Paula Castelões

**Affiliations:** 1 Intensive Care, Centro Hospitalar Vila Nova de Gaia/Espinho, Vila Nova de Gaia, PRT

**Keywords:** icu, intensive care unit, follow-up, readmission, critical care outcomes

## Abstract

Objective: Evaluate the impact of a post-discharge critical care transition program (CTP) on intensive care unit (ICU) readmission, in-hospital mortality, and six-month survival.

Methods: This was a prospective observational, single-center study, with a before-after design, in a critical care department in a tertiary hospital in Northern Portugal. Critically ill patients with ICU stay > 48 h or intermediate care stay >72 h or tracheostomized patients were included in the program. Historic controls included critically ill patients admitted in the six months prior to program implementation. The follow-up visit included a medical evaluation by an intensivist and a meeting with the attending physician. The primary outcome was critical care department readmission. Secondary outcomes were mortality at hospital discharge, 28-day, and six-month mortality. The readmission rate was compared between groups. Multivariate analysis and Kaplan-Meyer survival analysis were used to evaluate survival benefits.

Results: Between September 2020 and March 2021, 132 patients were included in the CTP. The Control group included 196 patients. The intensivist’s assessment led to management change in 15.1% of patients. The CTP group had a non-significant lower readmission rate (0.8% vs. 4.1%; p=0.09). Multivariate analysis showed a benefit for the CTP regarding in-hospital, 28-day, and six-month mortality. Kaplan-Meyer survival analysis showed improved survival in the CTP group.

Conclusions: The CTP reduced, non-significantly, the readmission rate, and significantly improved in-hospital and six-month mortality. Further analyses are needed to improve inclusion criteria and better allocate human resources.

## Introduction

The transition from an ICU to the general ward represents a challenge in the management of critically ill patients, with some patients being at high risk for readmission [1-2]. Multiple risk factors for ICU readmission have been identified, such as older age, higher comorbidity score, and longer ICU stay [3-4].

The subset of patients who deteriorate in the ward and need readmission have higher mortality rates than the general critical care population [5].

Different forms of CTPs have been implemented to improve the patient transition to the ward and lower readmission rates, most of which are critical care nurse-based [6- 7] with varying methodologies. These programs seem to provide a benefit in terms of readmission rate and in-hospital mortality [8-10], although limited by the quality of some of the data [11].

There are few studies [12-13] that explore the effectiveness of ICU-physician-based CTPs and the impact on outcomes.

Based on these results, we decided to implement an intensivist-based CTP in our hospital and to study its impact on the outcomes of critically ill patients (readmission rate and in-hospital survival).

## Materials and methods

Study design

This was a prospective interventional study of patients included in a critical care transition program (CTP) after discharge from the Critical Care Department (CCD), which functions in a closed ICU format. At the time of the study, the CCD had 12 ICU beds and another nine beds in a separate, high-dependency unit, for patients who require non-invasive mechanical ventilation or high-flow nasal oxygen therapy and where most critical care patients go through a step-down process before discharge to the ward. To better understand if the CTP had a positive effect on critical care patients’ outcomes, data from the CTP patients (cases) was compared with historic controls admitted in the previous six months (14th March to 15th September) before the implementation of the project, in a before-after analysis design. Data from the control group were obtained by reviewing medical electronic records.

The study period occurred between 15th September 2019 and 15th March 15th 2020, before a temporary suspension of the program due to the coronavirus disease 19 (COVID-19) pandemic. We present the results of the first six months of the program.

The main objective of the CTP was to promote early identification of deteriorating patients, in order to reduce readmissions, in-hospital and six-month mortality post-ICU stay. Readmission was defined as readmission to the CCD, for any reason, during the same hospitalization. For readmitted patients, data relative to the CCD [ICU stay, invasive mechanical ventilation (IMV) duration] was only counted for the first stay in the CCD. In-hospital mortality was defined as death, by any cause, during the same hospitalization. Mortality at 28-day and six-month were defined as death, by any cause, within the pre-specified time frame after the first CCD discharge. 

Demographic, clinical, and relevant outcome data were obtained prospectively by reviewing CTP evaluation records and medical records. Data from the control group were obtained by reviewing medical records. All data were stored according to ethical concerns and data protection laws.

Critical care transition program

Patients were eligible if they met one of the following criteria: ICU stay superior to 48 h or if the total CCD stay was longer than 72 h or if they were tracheostomized at discharge, regardless of the duration of ICU stay. Patients discharged to another hospital or discharged with a do-not-resuscitate order were excluded from the program.

The first follow-up visit was made between the first 24-72 h post-CCD discharge depending on the decision made by the intensivist at discharge. The need for further re-evaluations was assessed by the intensivist responsible for the follow-up consultation. Patients were evaluated during the weekdays, as the critical care transition team is not active during the weekend due to limited human resources. The patients were discharged from the CTP program when they presented no organ dysfunctions at re-evaluation or a decision to not readmit/not resuscitate was made based on clinical deterioration. During consultation, a full physical exam was performed. This examination included the application of several scales, namely visual analog scale for pain [14], Confusion Assessment Method (CAM-ICU) scale [15], Barthel Scale [16], and Glasgow Coma Scale (GCS).

After evaluating the patient, the intensivist discussed with the attending physician in the ward the clinical evolution of the patient and suggested prescription or management adjustments, including readmission to the CCD. The full protocol is available as supplementary data. The protocol and study design were reviewed and approved by “Comissão de Ética para a Saúde”, the local institutional review board (approval number 94).

Statistical analysis

Descriptive statistics were used to describe the study population characteristics at baseline. Discrete variables were presented as absolute frequencies with percentages. Continuous variables were presented as mean ± standard deviation, if normally distributed, otherwise as median ± (interquartile range).

A comparative analysis of intervention group (CTP group) and historic controls was performed regarding demographic and clinical data [Acute Physiology And Chronic Health Evaluation II (APACHE II) score; Simplified Acute Physiology Score (SAPS II) score; invasive mechanical ventilation duration, ICU length of stay; CCD readmission and in-hospital mortality, amongst others]. Variables were compared using the chi-square test or Fisher's exact test for categorical variables; parametric data were compared using the Students’ t-test and nonparametric data using the Mann-Whitney U test. Multivariate logistic regression analysis was performed to evaluate possible confounders for the different outcomes. Kaplan-Meyer methods were used to compare overall survival at hospital discharge, 28-day, and six-month post-CCD discharge between groups.

Data analysis was performed using Statistical Package for the Social Sciences (SPSS) 22.0 program (IBM Corp., Armonk, NY). Results were considered statistically significant for a two-sided p-value <0.05.

## Results

**Figure 1:**
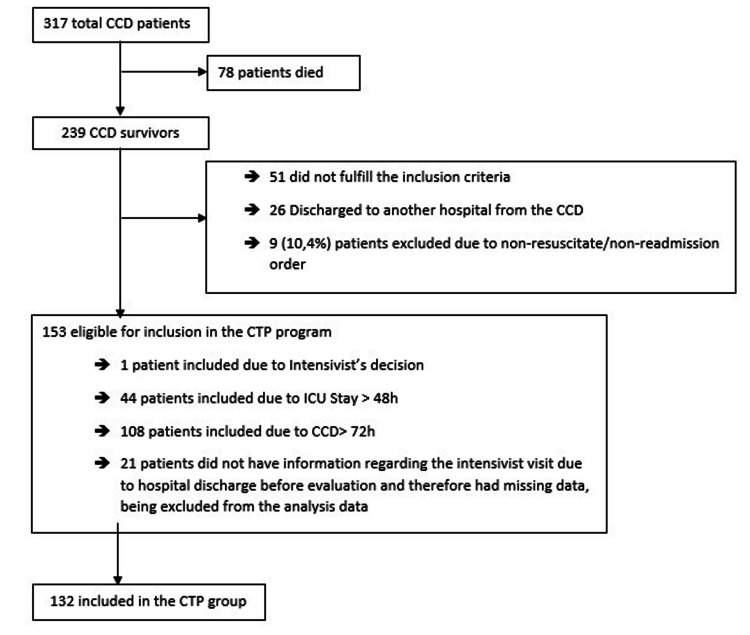
Patient flowchart. CTP, critical care training program; CCD, critical care department; ICU, intensive care unit

During the study period, 317 patients were admitted to the CCD and of these, 132 patients were included in the CTP group (Figure 1).

One patient died before the evaluation and the other 20 patients were discharged during the weekend. Regarding the historic control group, 196 patients were discharged from the CCD from 14th March to 14th September 2019 and included in the analysis.

Patients’ characteristics are displayed in Table 1.

**Table 1:** Population data. SD, standard deviation; IMV, invasive mechanical ventilation; CCD, critical care department; CTP, critical care transition program

	Control	CTP	p value
N	196	132	-
Age (mean ± SD)	62.36 ± 15.625	63.84 ± 16.89	0.415
Gender [male] n (%)	131 (66.8)	83 (62.9)	0.460
APACHE II (mean ± SD)	18.38 ± 7.366	18.41 ± 7.529	0.972
SAPS II (mean ± SD)	39.14 ± 15.407	41.61 ± 15.695	0.291
IMV n (%)	147 (75.0)	72 (54.5)	0.000
Duration of IMV (days), median (IQR)	5 (3-11)	6.5(4-13)	0.256
Duration of antibiotic therapy median - days (IQR)	4 (2-8)	3 (1-7)	0.031
Duration of CCD stay (days), median (IQR)	5.5 (3-10)	7 (4-12.5)	0.071
Level of care			0.059
Level II n (%)	73 (37.2)	63 (47.7)	-
Level III n (%)	123 (62.8)	69 (52.3)	-
Admission type			0.677
Medical n (%)	90 (45.9)	55 (41.7)	-
Elective surgical n (%)	42 (32.7)	28 (21.2)	-
Urgent surgical n (%)	64 (21.4)	49 (37.1)	-

Most patients were admitted for medical reasons. There were no differences in baseline characteristics, namely age, sex, severity scores, and CCD length of stay. The CTP group had a significantly lower percentage of patients that required invasive mechanical ventilation (72 vs. 147 patients, p<0.001) but without statistically significant differences in the duration of IMV.

Details from the follow-up visit are described in Table 2.

**Table 2:** Follow-up consultation data. VAS, visual analog scale; ICU-AW, intensive care unit-acquired weakness

Variable	Value
Time to Follow up visit	-
24 h n (%)	27 (20.5)
48 h n (%)	83 (62.9)
72 h n (%)	21 (15.9)
Other (>72 h) n (%)	1 (0.8)
ICU-AW n (%)	35 (26.5)
Barthel Scale median (IQR)	60,00 (25-100)
VAS > 0 n (%)	21 (15.9)
Tracheostomy n (%)	9 (6.8)
Dysphagia	
Liquids	7 (4.6%)
Mixed	8 (5.2%)
Patients with more than one follow-up visit n (%)	20 (15.2)
Intensivist action n (%)	20 (15.15)
Prescription change n (%)	14 (10.60)
New diagnostic workup n (%)	6 (4.55)
Time to hospital discharge (days), median (IQR)	8.5 (4-21)
Readmission n (%)	1 (0.75)
Hospital death n (%)	10 (6.5)

Most patients were evaluated 48 h post-discharge (62.9%) and 84.9% of all patients received only one follow-up consultation; 19 (14.4%) patients were visited twice and one patient required three visits before discharged from the CTP. During the first follow-up consultation, nine (6.8%) patients were tracheostomized and 70 patients (45.8%) were still completing their antibiotic course. Median Barthel Scale was 60 (25-100) and 13.7% of all patients had GCS<15 at follow-up, 15.9% of patients presented some level of pain according to visual analog scale (VAS). ICU-acquired weakness (ICU-AW) was diagnosed in 26.5% of patients.

Length of ICU stay (p<0.001) and duration of IMV (p<0.001) were significantly associated with ICU-AW at follow-up consultation.

Patient management was changed in 20 patients (15.1%) after the intensivists’ assessment (new diagnostic work-up - six cases; prescription change - 14 cases).

Admission due to urgent surgery was significantly associated with the need for medical intervention during the follow-up consultation (OR 3.18, 95% CI 1.19-8.43; p=0.01), as well as longer IMV duration (p=0.03) and higher VAS pain levels (p=0.03).

In the CTP group, six patients (4.5%) died before hospital discharge, and one was readmitted due to acute heart failure. Another six patients (4.5%) were discharged to a rehabilitation facility. 

The CTP group had non-significant inferior duration of stay until hospital discharge with significantly lower in-hospital (4.5% vs. 15.3; p<0.01) and six-month mortality (10.3% vs. 11.4%; p=0.01) (Table 3).

**Table 3:** Population comparison. CCD, Critical care department; CTP, Critical transition program

Variable	Control	CTP	p value
Time to hospital discharge (days) n (IQR)	12 (4-23.75)	8.5 (4-21)	0.264
CCD readmission n (%)	8 (4.1%)	1 (0.8%)	0.09
In-hospital mortality n (%)	30 (15.3)	6 (4.5)	0.000
28-day mortality n (%)	22 (11.2)	4 (3.0)	0.000
6-month mortality n (%)	19 (11.4)	13 (10.3)	0.01

Controlling for age, sex, the need for invasive mechanical ventilation and IMV duration, there was a protective effect for CTP regarding in-hospital (OR 0.250; p=0.004); 28-day (OR 0.235; p=0.012) and six-month mortality (OR 0.33; p=0.02) (Table 4). 

**Table 4:** Multivariate analysis. IMV, invasive mechanical ventilation; CTP, critical care transition program; OR, odds ratio; CI, confidence interval

Variable	In-hospital mortality			28-day mortality			6-month mortality			
	OR	95% C.I.	p value	OR	95% C.I.	p value	OR	95% C.I.	p value	
										IMV	0.760	0.296–1.949	0.567	0.635	0.204–1.977	0.433	0.480	0.215–1.074	0,074	
IMV duration	1.011	0.973–1.052	0.569	1.015	0.973–1.059	0,492	1.103	0.980–1.047	0.448	
Age	1.048	1.018–1.078	0.001	1.067	1.028–1.107	0.001	1.055	1.028–1.079	0.000	
Sex	1.121	0.527–2,382	0.767	0.231	0.074–0.718	0.011	1.150	0.616–2.146	0.660	
CTP	0.250	0.097–0.640	0.004	0.235	0.076–0.730	0.012	0.333	0.166–0.670	0.002	

**Figure 2:**
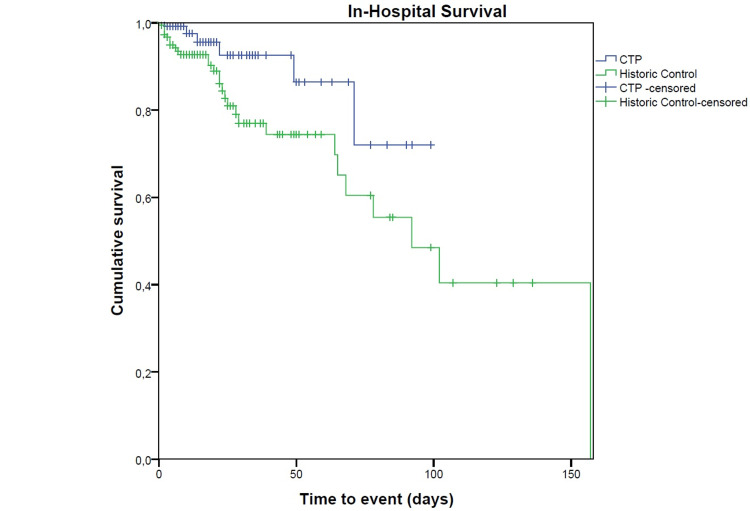
Kaplan-Meyer survival analysis for in-hospital survival. CTP, critical care transition program

**Figure 3:**
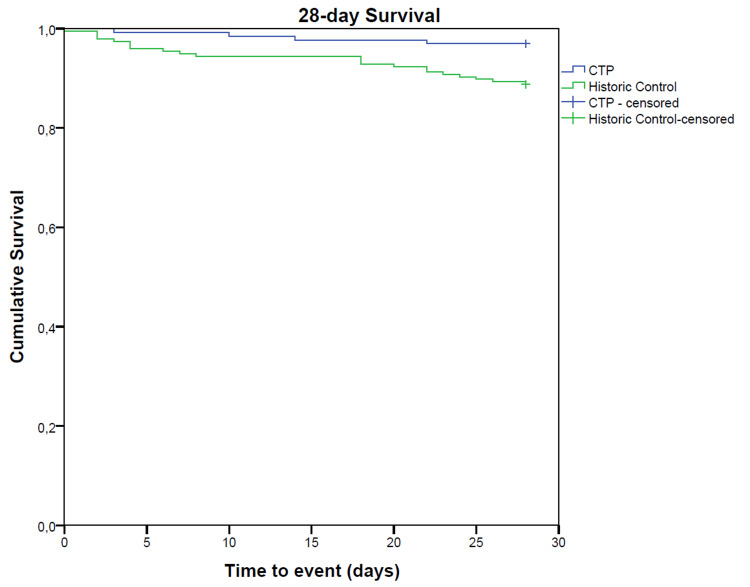
Kaplan-Meyer survival analysis for 28-day survival. CTP, critical care transition program

**Figure 4:**
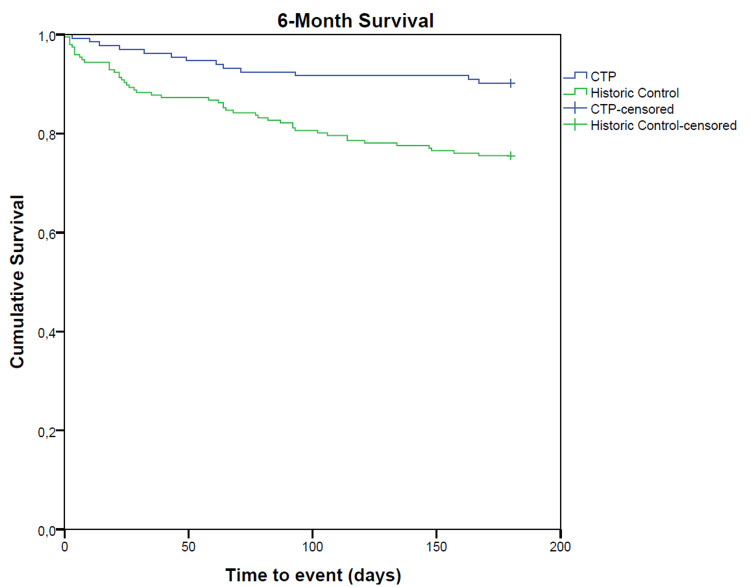
Kaplan-Meyer survival analysis for 16-month survival. CTP, critical care transition program

There was a significantly improved survival in the CTP group at hospital discharge, as well as 28-day and six-month post-CCD stay (Figures 2-4). 

Finally, regarding the 51 patients that were not included in the CTP during the study period, there were no readmissions and only one died in the hospital, an 89-year-old female patient initially admitted due to minor traumatic brain injury.

## Discussion

The rationale for CTPs stems from the need to improve handover between intensivists and other physicians, with the goal of improving in-hospital outcomes and reducing ICU readmission. Data regarding this type of interventions vary according to the setting and resources, ranging from lack of effect to lower readmission and mortality rates [5].

This study presents an analysis of an intensivist-based CTP and its impact on readmission rates and mortality.

The population in this study was a mixed medical and surgical population and there were no significant differences between groups except for the rate of IMV. Historic controls had higher rates of IMV (75% vs 54%, p<0.001). IMV is associated with worse outcomes, as a sign of higher severity of illness or as a risk for mortality, due to the need for deeper sedation and ventilator-induced diaphragm dysfunction [17]. Nevertheless, the difference in the rates of IMV reflects the progressive adoption of high-flow nasal oxygenation in our CCD, as suggested by recent guidelines [18]. There were no statistically significant differences regarding the duration of mechanical ventilation between historic controls and CTP patients (five vs six days, p=0.256), and multivariate analysis (Table 4) showed no impact of IMV on mortality. Furthermore, both groups had no differences between SAPS and APACHE II, indicating similar severity of illness.

There was a non-significant trend for readmission reduction after implementation of the CTP (0.8% vs 4.1%; p=0.09). The readmission rate for critical care patients reported in the literature varies from 4% [19] to 6%-7% for the mixed-ICU population [20-21]. In the historic control group, the readmission rate was similar to that of previous studies and the readmission rate in the intervention group was lower than previous reports despite strict inclusion criteria that selected the patients at highest risk for readmission. Based on these data, there could indeed be a positive effect from the critical transition program, but this study was not powered to show that difference. A larger sample size, may, in future studies, help to explore this difference.

Risk factors and readmission rates have been extensively studied through the years and there are conflicting data regarding the use of readmission report as a quality indicator of ICU quality of care [22-23]. The long-term mortality of both groups was evaluated to understand whether a smoother transition to the ward could have an impact on the overall survival at long-term post-discharge from the critical care setting.

There was a benefit in intra-hospital mortality and six-month mortality after the implementation of the CTP (Table 3). A recent review of CTPs [9] identified a non-significant trend for better survival when this type of programs is implemented. In this study, this benefit was consistent when using logistic regression analysis to control for gender, need for IMV, and duration of IMV. Older age was the only other factor associated with long-term mortality.

In 15.1% of cases the assessment by the attending intensivist led to a direct change in patient management. Pain medication optimization was one of the goals of the intensivist. The median Barthel Scale in the CTP group (60 points) and intensive care unit-acquired weakness (ICU-AW) (26.5%) rates reflects the frailty of critical care patients after the acute illness episode.

 These results highlight the complexity of a critical care patient after discharge to the ward, whether due to a direct impact from the disease that caused ICU admission or due to sequelae from the organ support (i.e., tracheostomy due to prolonged weaning after IMV, delirium, ICU-AW). The complex physiology and the significant decrease in clinical monitoring have been associated with clinical deterioration and mortality in previous studies [24]. Inadequate and incomplete handover information have also been associated with the deterioration of critical care patients after the transition to the ward [25]. This CTP program tackles this difficulty by ensuring that one of the goals of the evaluation is a meeting between the intensivist and the attending physician. A smoother transition to the general ward, with the improvement of the communication and cooperation between intensivists and other physicians, may have had an impact on the long-term outcomes (in-hospital and six-month mortality), as reported in other studies [11]. There are also qualitative reports that show some emotional benefit [26], enabling the patient to emotionally cope with the sequelae from the ICU stay.

Further refinement of the inclusion criteria could help to better allocate human resources, ensuring that only patients with the highest risk for readmission are evaluated by the medical team. Expanding the team, by including an ICU liaison nurse, respiratory therapist, and a physiotherapist could improve the benefits of this program. Furthermore, CTPs are only one strategy in the step-down of care from ICU patients, mainly providing support during the hospital stay. These must be complemented by post-discharge strategies, such as follow-up clinics [27-28], in order to maximize adequate support and to ensure adequate transition and quality-of-life focused care.

Most CTPs data come from the highest income countries such as the United Kingdom (UK) and Australia (9), with a higher rate of critical care bed per capita [29-30] such as 6.6 beds per 100000 inhabitants in the UK or 8.94 beds in Australia vs 4.2 beds per 100000 inhabitants in Portugal. These results show that CTPs are feasible and effective even in different macroeconomical scenarios, with strict inclusion criteria that maximize resource management.

This intensivist-based CTP was systematically performed by an experienced medical team, with a positive impact on long-term outcomes which validates the methodology of this study. The lack of readmissions and reduced mortality in the patients excluded from the CTP reinforce the accuracy of the inclusion criteria.

Small sample sizes and single-center data may compromise the generability of the results. Furthermore, any CTP program must be adapted within the local hospital framework and resources available. The non-randomized study design poses a risk for selection bias.

## Conclusions

A structured intensivist-led short-term follow-up and CTP showed a non-significant trend towards lower ICU readmission rate and a positive impact on in-hospital and six-month mortality.

Our eligibility criteria successfully identified patients at higher risk of deterioration. This CTP is a feasible option that ensures a smoother transition to the general ward, with improved communication and cooperation between intensivists and other physicians. Further studies with a larger sample size are needed to study the impact on readmission rates.

## Appendices

Critical care transition program protocol

Patients were eligible to be included in the critical care transition program (CTP) if their intensive care unit (ICU) stay was superior to 48h or if the total critical care department (CCD) stay was >72 h. Tracheostomized patients were also included in the follow-up program. Patients discharged to another facility and discharged with a “Do not resuscitate” order were excluded from the program. A follow-up visit was made at 24/48/72 h according to the medical team availability. Re-evaluation was defined by the intensivist responsible for the visit.

Patients were deemed fit for discharge from the CTP when they presented no organ dysfunction at re-evaluation or a change in goals of care was made (i.e., do not resuscitate/do not readmit in intensive care).

During the consultation, a full physical exam must be performed, including an organ and system revision. Several scales must also be applied during the consultation, including the Visual Analog Scale (VAS) for pain, the Confusion Assessment Method-ICU (CAM-ICU) scale, the Barthel Scale, and the Glasgow Coma Scale (GCS).

After consultation, the intensivist should meet the attending physician at the ward, to evaluate and, if necessary, re-evaluate the treatment goals. If needed, readmission to the CCD must also be decided at this point in time. Readmission was only counted if patients were readmitted, by any motive, during the same hospitalization.

Inclusion Criteria

1- ICU stay >48 h or high-dependency unit >72 h

2- Total stay in Intensive Care Department >72 h

3- Tracheostomy status for ventilation weaning/airway protection in neurocritical care patients

4- Nocturnal non-invasive ventilation support

5- ICU acquired weakness at discharge

6- Delirium (hypoactive/hyperactive)

7- Decision by intensivist at discharge from the CCD (must disclose the specific reason for follow-up)

Exclusion Criteria

1- Non-readmission decision at discharge/Discharge in the setting of palliative care

2- Discharge to another facility

Re-evaluation Criteria

1- Further re-evaluation could be planned according to the decision of the intensivist’s first decision

2- Weekly re-evaluation for tracheostomized patients and a final re-evaluation 24 h post-decannulation

Criteria for Follow-Up Discharge

1- No organ disfunction at re-evaluation

2- New decision to not readmit the patient in the ICU at discharge

3- Tracheostomy decannulation (if follow-up due to tracheostomy status)

Intensivist Visit

The visit, by the intensivist, at the ward, consists of

-> Full physical exam was performed, including an organ and system revision

-> Severity score and scales application [Visual Analog Scale (VAS) for pain, Confusion Assessment Method-Intensive Care Unit (CAM-ICU) scale, Barthel Scale, Glasgow Coma Scale (GCS)].

-> Reunion with the attending physician in the ward, to evaluate and discuss possible diagnostic and management changes, required level of care and prognosis and, if needed, readmission to the CCD was also decided at this point.

Evaluation Timing

- Patients received follow-up consultation at approximately 48 h post-discharge, as a general rule. A longer time for re-evaluation was warranted if the patient were discharged on Friday (evaluation on Monday). Shorter time to the consultation was also warranted, either by the intensivist's decision at discharge from the ICD or in cases when the patient was discharged on Thursday (consultation 24 h later, on Friday).

Readmission Decision

If a patient, during the medical evaluation, has organ dysfunction that requires monitoring and treatment in a higher level of care than the ward, the CCP physician would contact the lead assisting physician in the CCD, on that day, to proceed with readmission in the CCD.
